# Deep Learning Artificial Intelligence to Predict the Need for Tracheostomy in Patients of Deep Neck Infection Based on Clinical and Computed Tomography Findings—Preliminary Data and a Pilot Study

**DOI:** 10.3390/diagnostics12081943

**Published:** 2022-08-12

**Authors:** Shih-Lung Chen, Shy-Chyi Chin, Chia-Ying Ho

**Affiliations:** 1 Department of Otorhinolaryngology and Head and Neck Surgery, Chang Gung Memorial Hospital, Linkou 333, Taiwan; 2 School of Medicine, Chang Gung University, Taoyuan 333, Taiwan; 3 Department of Medical Imaging and Intervention, Chang Gung Memorial Hospital, Linkou 333, Taiwan; 4 Division of Chinese Internal Medicine, Center for Traditional Chinese Medicine, Chang Gung Memorial Hospital, Taoyuan 333, Taiwan

**Keywords:** artificial intelligence, deep learning, deep neck infection, tracheostomy

## Abstract

**Background:** Deep neck infection (DNI) can lead to airway obstruction. Rather than intubation, some patients need tracheostomy to secure the airway. However, no study has used deep learning (DL) artificial intelligence (AI) to predict the need for tracheostomy in DNI patients. Thus, the purpose of this study was to develop a DL framework to predict the need for tracheostomy in DNI patients. **Methods:** 392 patients with DNI were enrolled in this study between August 2016 and April 2022; 80% of the patients (n = 317) were randomly assigned to a training group for model validation, and the remaining 20% (n = 75) were assigned to the test group to determine model accuracy. The *k*-nearest neighbor method was applied to analyze the clinical and computed tomography (CT) data of the patients. The predictions of the model with regard to the need for tracheostomy were compared with actual decisions made by clinical experts. **Results:** No significant differences were observed in clinical or CT parameters between the training group and test groups. The DL model yielded a prediction accuracy of 78.66% (59/75 cases). The sensitivity and specificity values were 62.50% and 80.60%, respectively. **Conclusions:** We demonstrated a DL framework to predict the need for tracheostomy in DNI patients based on clinical and CT data. The model has potential for clinical application; in particular, it may assist less experienced clinicians to determine whether tracheostomy is necessary in cases of DNI.

## 1. Introduction

Deep neck infection (DNI) affects the fascial spaces of the neck and can be fatal [1]. DNI may cause airway compromise, which is associated with serious morbidity and even mortality. To manage DNI, protecting the airway is essential [2]. Tracheostomy is considered for DNI patients when intubation is hard to perform. However, whether to perform tracheostomy usually depends on the physician’s clinical consideration.

Artificial intelligence (AI) allows computers to perform tasks that normally require human intellect and cognitive processes [3]. Machine learning is a form of AI that allows predictions to be made based on information extracted from input data [4,5,6]. Multilayered architecture based on mathematical functions allows machines to learn and think more deeply, and to interpret complex data in a highly precise manner. Such machine learning methods are referred to as deep learning (DL). DL AI has made remarkable progress in recent years [7]. However, to date no DL model is available to help physicians determine when to perform tracheostomy in cases of DNI, especially when there is no obvious sign of airway obstruction. Thus, our goal was to establish a DL model for predicting the need for tracheostomy in patients with DNI.

## 2. Materials and Methods

Between August 2016 and April 2022, this study involved a retrospective review of the medical records of 392 DNI patients admitted to Chang Gung Memorial Hospital in Linkou, Taiwan. Computed tomography (CT) was performed for diagnostic imaging. When the DNI cause the airway obstruction, progression of symptom was observed in the DNI after 2 days of intravenous antibiotics using, or ≥2 cm abscess was detected, the incision and drainage was performed.

According to patient’s vital signs, blood oxygen saturation, respiratory situation, laboratory and imaging findings, the treating physician decided whether each patient should undergo tracheostomy to secure the airway [8].

Ceftriaxone (1 g, q12 h) and metronidazole (500 mg, q8 h) were the empiric antibiotics [9]. The antibiotic regime can be adjusted depending on the pathogen culture. If no clear microorganisms are recognized, patients are treated with intravenous antibiotics for 7–10 days, followed by 7 days of oral amoxicillin trihydrate + clavulanate potassium or clindamycin [10].

### 2.1. Measurement of CT

**Figure 1:**
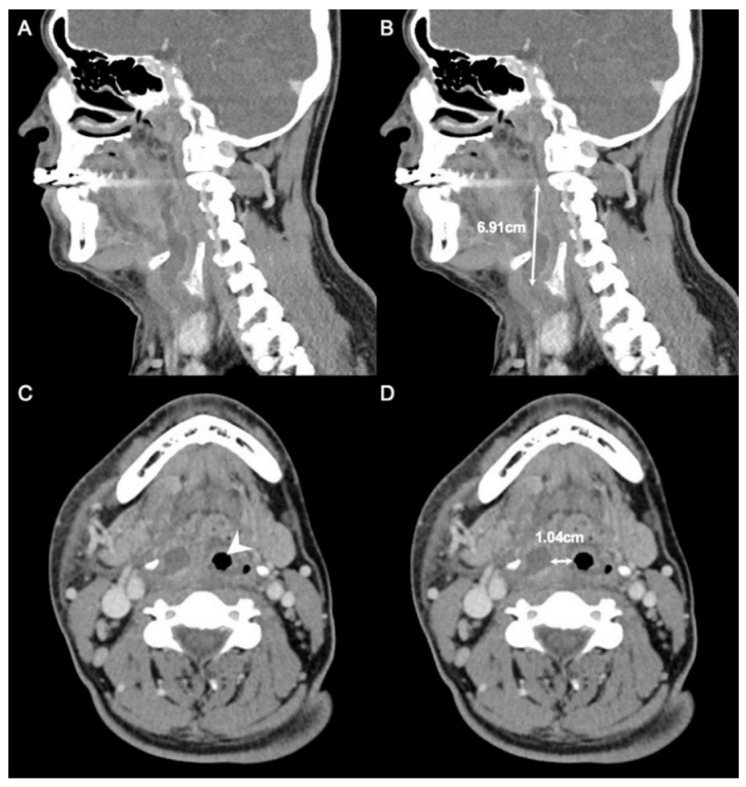
Parameters measured on computed tomography (CT) scans. (**A**,**B**) The maximum diameter of the abscess was determined based on axial, coronal, and sagittal CT scans. (**C**,**D**) The distance between the abscess and upper airway inlet was measured on axial scans. Arrowhead, upper airway inlet; double arrow, distance measured on CT scans.

We measured the maximum diameter of the abscess in an axial, coronal, or sagittal CT scan. Next, we measured the nearest distance from abscess to the inlet of the trachea on the axial scan; both measurements were used as DL parameters (Figure 1A–D).

### 2.2. Data Collection

To establish the DL model for predicting the need for tracheostomy, we collected the following clinical data based on medical records as Table 1 shown. Together with the maximum diameter of the abscess and the nearest distance from the abscess to the inlet of trachea, these clinical variables were entered into the DL model. The values for all continuous and categorical variables were standardized, i.e., were converted into *z*-scores. We subtracted the mean score for a given variable from all individual scores and then divided the remainder by the standard deviation [11].

### 2.3. k-Nearest Neighbor Method

To develop a DL model, the dataset of interest is first separated into training and test subsets [4,6]. The model can then be validated using the test dataset; this allows for the accurate prediction of model performance when analyzing previously unseen data [3].

**Figure 2:**
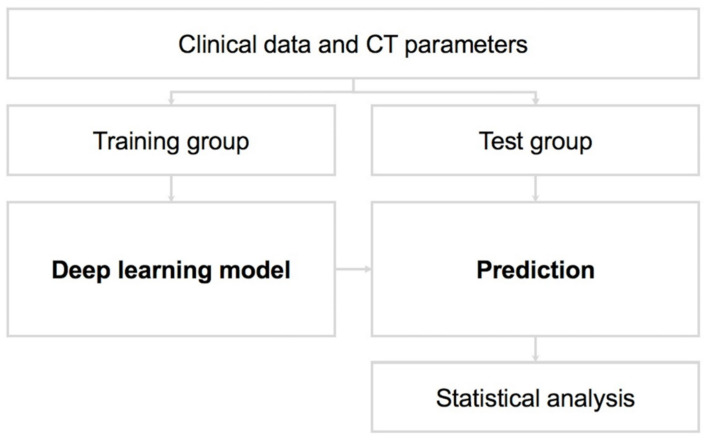
Training and test datasets for the deep learning model.

**Figure 3:**
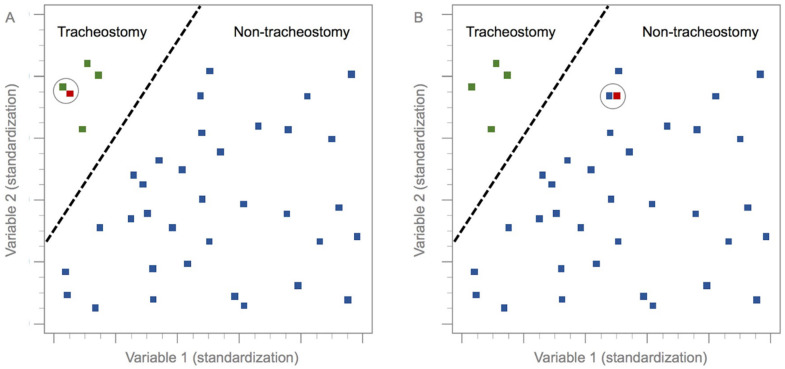
Diagram of the *k*-nearest neighbor model. In (**A**,**B**), green dots represent training group patients who underwent tracheostomy, and blue dots represent training patients who did not undergo tracheostomy. Red dots represent test group patients. The dotted line distinguishes cases in which tracheostomy was performed from those in which it was not performed. The circles are the nearest neighbors to test and training group instances.

In this study, 80% of the data (n = 317) were randomly selected for model training; the remaining 20% (n = 75) were used for testing the model (Figure 2). Several mathematical algorithms may be used for DL models; the *k*-nearest neighbor (*k*-NN) method was used for this DL model. The *k*-NN algorithm is used to classify hitherto unclassified data, based on the classification of the nearest neighbors among a set of previously classified instances [12,13,14,15,16]. In other words, the *k*-NN algorithm measures the distance or similarity between test and training instances [17,18,19], and classifies each training set instance based on its similarity to its neighbors. The final classifications and output depend on the distances between the test and training data (Figure 3) [5,6,11,14,20,21].

When using the *k*-NN algorithm, Euclidean distance *D* is obtained to represent the distance between two points, *x* and *y*, in n-dimensional space, with each n-dimension corresponding to one of the n-features needed to characterize an instance [11,19,22,23]. The following formula is used:

$$D\left(x,\;y\right)=\sqrt{{\left(x1-y1\right)}^{2}+{\left(x2-y2\right)}^{2}\ldots +{\left(xn-yn\right)}^{2}}$$

The *k* value used should be that resulting in the highest classification accuracy [19]. In this study, *k* = 1 was chosen because this value provided the optimal classification performance after cross-validation, as the previous study [21].

After verifying our model, we used it to predict the need for tracheostomy in DNI patients. The model parameters were optimized through an iterative process that progressively reduced the discrepancy between the actual and expected model outputs [6].

### 2.4. Exclusion Criteria

Patients with immunocompromised status, serious cardiopulmonary illness, or history of head and neck trauma were excluded. In total, 392 patients were enrolled.

### 2.5. Statistical Analysis

The Kolmogorov–Smirnov test revealed that the data were not normally distributed, so we used the chi-square and Mann–Whitney *U* tests to analyze categorical and continuous variables, respectively. Classification accuracy (tracheostomy vs. non-tracheostomy) was calculated as the ratio between the number of correctly classified patients and the total number of patients [11]. Sensitivity (true-positive rate) refers to the proportion of correctly identified positive (tracheostomy) patients, while specificity (true-negative rate) is the proportion of correctly identified negative (non-tracheostomy) patients. All data were analyzed using MedCalc software (ver. 18.6; MedCalc, Ostend, Belgium) and Excel (Microsoft Corp., Redmond, WA, USA) [7,24]. A *p* value < 0.05 was considered to reflect statistical significance.

## 3. Results

Table 1 lists demographic and clinical data. In total, 392 patients with DNI were enrolled: 261 males (66.58%) and 131 females (33.42%) with a mean age of 51.36 ± 18.74 years. The mean chief complaint period was 5.04 ± 4.49 days. With regard to laboratory data, the mean WBC count was 15,007.39 ± 5801.19 μL, the mean CRP level was 156.94 ± 99.61 mg/L, and the mean blood sugar level was 142.66 ± 72.46 mg/dL. Furthermore, 147 (37.50%) patients had DM status.

Involvement of single deep neck space was observed in 108 (27.55%) patients, while double spaces were involved in 151 (38.52%) patients, and three or more spaces were involved in 133 (33.93%) patients. Mediastinitis was observed in 20 (5.10%) patients. On CT images, the mean maximum diameter of abscess was 6.36 ± 3.08 cm, and the mean nearest distance from abscess to inlet of trachea was 1.41 ± 1.35 cm. A tracheostomy was performed in 50 (12.75%) patients.

Table 2 compares the 317 patients in the training group with the 75 patients in the test group. No significant differences were observed between the two groups in terms of clinical variables or CT scan parameters.

Based on the parameters which we chose, our DL model yielded a patient classification accuracy of 78.66% (59/75). The analysis revealed that the sensitivity and specificity values were 62.50% and 80.60%, respectively.

## 4. Discussion

Complications of DNI can include esophageal perforation, pneumonia, internal jugular vein thrombosis (Lemierre’s Syndrome), carotid artery erosion, and airway compromise [25,26,27]. The mortality rate is relatively high whiles these complications occur [28]. A tracheostomy is needed in some DNI cases to secure the airway.

DL models are used for making predictions based on previous observations [6,29]. Several DL algorithms are available to analyze large datasets; through such analyses, complex and heterogeneous data can inform real-world clinical practice and recommendations [30,31,32,33,34]. The medical applications of DL include cancer diagnosis, prognostic predictions, integration of clinical and genomic data, clinical trial design, and analysis of readmission and mortality data [35,36,37,38,39]. With regard to infectious diseases, DL has been used to aid diagnosis, predict severity, and determine the most appropriate antimicrobial treatment for individual patients [40]. Wilson et al. used DL to diagnose peritonsillar abscess with high accuracy [4]. Our DL model was able to predict whether tracheostomy would be needed for DNI patients based on their clinical and CT data; the results suggest that it could be used in clinical practice.

The *k*-NN algorithm is one of the oldest, simplest, and most accurate DL algorithms for data mining and pattern classification, and is widely applied in many fields [17,21,41,42,43]. The *k*-NN algorithm operates on the assumption that instances in a dataset are often in close proximity to other instances with similar characteristics; classification is based on the similarity of instances with their nearest neighbors. The relative distance between instances is more important than their absolute position within a given region [19]. The *k*-NN algorithm is suitable for analyzing large, multidimensional datasets [41,44], and is the optimal method when prior knowledge of the data distribution is lacking [17,45]. Furthermore, there is no requirement for off-line training when using the *k*-NN algorithm, so it is also time efficient [14]. It already plays an important role in the fields of transportation, information security, and medicine [21].

As a user-defined integer, the value of *k* is typically small. If *k* = 1, the algorithm considers the nearest neighbor to be an unclassified instance. If *k* = 3, *k*-NN compares the distance to the unclassified instance among its three nearest neighbors [11]. When small *k* values are used, approximation error decreases while estimation error increases; the opposite trends are seen when *k* takes a large value. In practical applications, *k* generally takes a relatively small value, and cross-validation is usually used to determine the most appropriate value [21]. The 1-NN classifier is usually used as a benchmark for other classifiers because it exhibits reasonable performance for many pattern classification problems [14].

In this research, most patients were males, and this preponderance has been detected in former reports [9,46]. The average age of our patients was middle age, which was consistent with the prior researches [47,48]. Only significant factors can be used for classification [23], and research is ongoing to determine how to identify the most important variables and features for learning algorithms [49,50,51]. In this study, factors were selected for the DL model based on the ease of implementation and interpretation, with the goal of providing clinicians with insight into the circumstances under which tracheostomy should be performed. We considered the maximum diameter of the abscess, and its distance from the upper airway inlet on CT scans, to be the most influential parameters with regard to the decision to perform tracheostomy. Therefore, we included these two CT parameters in the training model.

As shown in Table 2, no significant differences were observed in clinical variables or CT parameters between the training and test groups. As with other DL models, we input retrospective data, such that the model was based on the past decisions of clinicians. Our DL model yielded a prediction accuracy of 78.66%. Failure to achieve a better accuracy may have been related to the variables used in the model, and to the subjective nature of clinicians’ decisions to perform tracheostomy. We did not consider the reason why DL is necessary because of the increasing errors of physicians’ clinical judgment. Conversely, this DL model can help clinicians determine whether patients should undergo tracheostomy at the beginning of the treatment course; this could be especially valuable for physicians who are less experienced in making decisions about whether to perform tracheostomy. Well-designed models with acceptable prediction accuracy based on training data can be tuned to handle new data inputs [6].

### Study Limitations

Limitations of this study included the use of retrospective data, reliance on patient self-reports for medical history data, subjective judgment, and decision making for tracheostomy, and manual measurement of CT scans. Thus, the disparities or inconsistencies could occur due to these biases. This pilot study is preliminary research, which has several deficits to address. Furthermore, the dataset was also relatively small (n = 317 in training group; n = 75 in test group) and based on a single institution.

## 5. Conclusions

We demonstrated a DL model to predict the need for tracheostomy based on patients’ clinical and CT data. It can help clinicians to decide whether tracheostomy should be performed in cases of DNI, and may lead to improvements in critical care.

## Figures and Tables

**Table 1:** Clinical characteristics of the 392 patients with deep neck infection.

Characteristics	N (%)
Gender	392 (100.0)
Male	261 (66.58)
Female	131 (33.42)
Age, years ± SD	51.36 ± 18.74
Chief complaint period, days ± SD	5.04 ± 4.49
WBC, uL ± SD	15,007.39 ± 5801.19
CRP, mg/L ± SD	156.94 ± 99.61
Blood sugar, mg/dL ± SD	142.66 ± 72.46
Diabetes mellitus	147 (37.50)
Deep neck infection space involved	
Single space	108 (27.55)
Double spaces	151 (38.52)
Multiple spaces, ≥3	133 (33.93)
Mediastinitis	20 (5.10)
Maximum diameter of abscess, cm ± SD	6.36 ± 3.08
Nearest distance from abscess to inlet of trachea, cm ± SD	1.41 ± 1.35
Tracheostomy performance	50 (12.75)

N, numbers; SD, standard deviation; WBC, white blood cell (normal range: 3500–11,000/μL); CRP, C-reactive protein (normal range < 5 mg/L); Blood sugar (normal range: 70–100 mg/dL). Maximum diameter of abscess and nearest distance from abscess to inlet of trachea were evaluated in CT scan.

**Table 2:** Clinical and computed tomography data of the training and test groups.

Characteristics	Training Group; N (%)	Test Group; N (%)	*p* Value
Gender	317 (100.0)	75 (100.0)	
Male	215 (67.82)	46 (61.33)	0.340
Female	102 (32.18)	29 (38.67)	
Age, years ± SD	50.88 ± 18.89	53.40 ± 18.06	0.364
Chief complaint period, days ± SD	5.20 ± 4.79	4.34 ± 2.79	0.455
WBC, μL ± SD	14,824.91 ± 5732.75	15,778.66 ± 6060.84	0.240
CRP, mg/L ± SD	155.08 ± 98.23	164.81 ± 105.52	0.511
Blood sugar, mg/dL ± SD	140.51 ± 70.13	151.77 ± 81.46	0.080
Diabetes mellitus			0.598
Yes	121 (38.17)	26 (34.66)	
No	196 (61.83)	49 (65.34)	
Deep neck infection space involved			
Single space	92 (29.02)	16 (21.33)	0.198
Double spaces	120 (37.85)	31 (41.33)	0.599
Multiple spaces, ≥3	105 (33.13)	28 (37.34)	0.499
Mediastinitis			0.557
Yes	15 (4.73)	5 (6.66)	
No	302 (95.27)	70 (93.34)	
Maximum diameter of abscess, cm ± SD	6.23 ± 2.91	6.92 ± 3.71	0.293
Nearest distance from abscess to inlet of trachea, cm ± SD	1.49 ± 1.44	1.03 ± 0.79	0.169
Tracheostomy performance			0.700
Yes	42 (13.24)	8 (10.66)	
No	275 (86.76)	67 (89.34)	

N, numbers; SD, standard deviation; WBC, white blood cell (normal range: 3500–11,000/μL); CRP, C-reactive protein (normal range < 5 mg/L); Sugar (normal range: 70–100 mg/dL). Maximum diameter of abscess and nearest distance from abscess to inlet of trachea were evaluated in CT scan.

## Data Availability

All data generated or analyzed in the study are included in this published article. The data are available on request.

## Abbreviations

AI: artificial intelligence; CT, computed tomography; CRP, C-reactive protein; DM, diabetes mellitus; DNI, deep neck infection; DL, deep learning; WBC, white blood cell.
